# ICU-acquired infection in neutropenic patients

**DOI:** 10.1186/s13054-026-05890-5

**Published:** 2026-03-17

**Authors:** Océane Bernard De Lajartre, Nicolas Massart, Florian Reizine, Anaïs Machut, Charles-Hervé Vacheron, Anne Savey, Arnaud Friggeri, Alain Lepape

**Affiliations:** 1 Service de Réanimation Polyvalente, Centre Hospitalier de Saint Brieuc, 10, Rue Marcel Proust, 22000 Saint-Brieuc, France; 2 Service de Réanimation Polyvalente, Centre Hospitalier de Vannes, Vannes, France; 3 REA-REZO Infections Et Antibiorésistance en Réanimation, Hôpital Henry Gabrielle, Saint-Genis-Laval, France; 4 Département d′Anesthésie Médecine Intensive Réanimation, Centre Hospitalier Lyon Sud, Hospices Civils de Lyon, 165 Chemin du Grand Revoyet, 69310 Pierre-Bénite, France; 5 PHE3ID, Centre International de Recherche en Infectiologie, Institut National de La Santé Et de La Recherche Médicale U1111, CNRS Unité Mixte de Recherche 5308, École Nationale Supérieure de Lyon, Université Claude Bernard Lyon 1, Villeurbanne, France; 6 Service de réanimation médicale et maladies infectieuses, CHU Rennes, 2 rue Henri Le Guilloux, 35000, Rennes, France

**Keywords:** ICU-acquired infections, Blood stream infection, Catheter line-associated blood stream infection, Ventilated associated pneumonia, Neutropenia

## Abstract

**Background:**

Among neutropenic patients, sepsis is a frequent cause of ICU admission, and its epidemiology is well described, but data on infection acquired during the ICU stay (ICU-AI) are lacking.

**Aim:**

We aim to describe, using a large nationwide cohort study, the incidence of ICU-AI for neutropenic patients in a large network of French intensive care units (ICUs).

**Methods:**

This study was conducted using the healthcare-associated infection surveillance cohort “REA-REZO” involving 265 participating ICUs. All patients admitted to a participating ICU from 1st January 2018 to 31th December 2023, for more than 48 h were eligible. The primary endpoint was the rate of ICU-AI. A matched analysis was conducted using non-parsimonious regression model to compare neutropenic and non-neutropenic patients.

**Findings:**

Matching process resulted in well-balanced 7545 patients’ pairs. ICU-AI incidence rate was 14.9 versus 13.0 AI per 1000 patients days in the neutropenic group vs non-neutropenic group (Incidence Rate Ratio [IRR] = 1.15 [1.05, 1.25] p = 0.002) with higher incidence rate for bloodstream infection (BSI) (p < 0.001) but comparable incidences rates of pneumonia (p = 0.503) and central-line associated BSI (p = 0.100). There were less enterobacteral*es* among ICU-AI of the neutropenic group (33.8% vs 45.4%, p < 0.001)**,** but higher rate of coagulase negative *Staphylococcus (*12.0% vs 5.2%, p < 0.001), *Enterococcus spp,* (8.9% vs 6.0%, p = 0.015) and *Candida spp*. (10.4% vs 6.2%, p = 0.001). Rates of ICU-AI involving multidrug resistant micro-organisms did not differed, neither did the rate of ICU-AI involving non fermenting Gram-negative bacilli.

**Conclusion:**

Neutropenic patients had higher ICU-AI incidence as compared with non-neutropenic patients, specifically through an increase of BSI incidence. Microbiology differs between neutropenic patients and non-neutropenic patients with no difference in multidrug resistant microorganism involvement.

**Supplementary Information:**

The online version contains supplementary material available at 10.1186/s13054-026-05890-5.

## Introduction

While immunocompromised patients account for an increasing proportion of ICU admissions, their outcomes remain poor [1-3]. This population is highly heterogeneous, and accumulating evidence suggests that infectious risk, clinical presentation, microbiological patterns, and outcomes differ substantially across subgroups of immunosuppression [3, 4].

Neutropenic patients represent a frequent and clinically distinct subgroup of other immunocompromised patients requiring frequent ICU admission for sepsis [5]. Frequent hospitalizations and repeated or prolonged antimicrobial exposure modify their microbiological ecology, favoring colonization and infection with resistant pathogens [6].

Recent studies in immunocompromised patients have challenged the notion of an increased risk of ICU-acquired infections in this population. However, these studies did not specifically focus on neutropenic patients [3, 4]. Neutropenic patients are particularly vulnerable to opportunistic infections, with an increased risk of invasive fungal and viral infections [7-10]. While early management has been extensively described for neutropenic patients [5], and despite their specificities, the impact of neutropenia on ICU-acquired infections and microbial ecology has not been adequately evaluated in large cohorts.

We therefore aimed to describe, using a large nationwide cohort, the incidence of ICU-acquired infections in neutropenic patients admitted to a broad network of French ICUs.

## Materiel and methods

### Patients and setting

This study was conducted using the REA-REZO prospective continuous multicenter cohort. It was a healthcare-associated infection surveillance network with collection of patient-level data of all adult patients hospitalized for at least 2 calendar days in any of the 265 contributing ICUs of the REA-REZO network since January 2018. Surveillance focused on ICU-AI and was discontinued when patients either died or were discharged from ICU. The detailed protocol for data collection and monitoring was available at: https://rearezo.chu-lyon.fr/.

In the present study, all patients admitted to a participating ICU from 1 st January 2018 to 31th December 2023, for more than 48 h were eligible, at the exception of those with liberty deprivation (by administrative decision *i.e.* guardianship), and patients younger than 18 years old who were excluded from the study. Follow-up was pursued until ICU discharge or death.

### Definition

Neutropenia was defined by polynuclear neutrophil counts < 0.5 G/L upon admission. There were no follow up of neutropenic count during ICUs stay and so patients who developed neutropenia during ICU stay were included in the non-neutropenic group.

Infection was considered as ICU-acquired if diagnosed 48 h after ICU admission and not incubating on admission [11]. The ICU-AI were diagnosed by the treating physicians.

Three distinct ICU-AI were considered for the present study: bloodstream infection (BSI), central line-associated bacteremia (CLABSI) and ICU-acquired pneumonia (ventilator associated or not). BSI was defined as a positive blood culture occurring 48 h or more after admission. Regarding common skin contaminants, 2 positives blood cultures drawn on separate occasions were required [12]. CLABSI was defined by the presence of a pathogen in a blood culture (a single blood culture for an organism not commonly found on the skin, and 2 or more blood cultures for an organism commonly found on the skin) associated with a positive catheter tip culture with the same microorganism in a patient who had a central line at the time of infection, or within 48 h prior to the onset of infection. Positive catheter tip culture without positive blood culture were not considered in the present analysis [13]. In patients with positive blood culture but in whom no catheter tip culture was performed were considered as having bloodstream infection. Differential time to positivity was not collected in the present study.

The diagnosis of pneumonia was based on a body of argument including clinical signs (fever, purulent sputum, hypoxia), radiological findings (new infiltrate), and leukocytosis. Ventilator-associated pneumonia (VAP) was defined as a pneumonia that arises more than 48 h after mechanical ventilation while ICU acquired pneumonia was considered in other cases if infection occurred more than 48 h after admission [14]**.** Microbiological documentation was performed by positive culture on respiratory samples (tracheal aspiration or bronchoalveolar lavage, protected specimen brush).

Microorganisms responsible for infection were considered as multidrug resistant micro-organisms (MRDO) according to the European Society of Clinical Microbiology and Infectious Disease definition [15].

Systemic antibiotic at ICU admission was defined as a curative antibiotic for a suspected or confirmed infection within 24 h before or after admission. Turn-over estimated ICU overload by year and was calculated as follow: number of admissions by year/number of ICU bed in the unit of admission.

Hydro-alcoholic solution (HAS) consumption (L/patient/year) was used as an indicator of hygiene quality. The HAS consumption was calculated with the consumption of HAS in one year divided by the number of days of hospitalization in the same ICU within the same year.

Pandemic period is defined by the period after March 2019 to December 2020. The region of admission was defined according to the hospital at the time of admission to intensive care. A rectal colonization surveillance was conducted in 103 out of 265 participating ICUs. In these ICUs, the patients were screened for MDRO rectal carriage at ICU admission, weekly afterwards and at discharge on rectal swabs. As described elsewhere, the patients with no prior colonization (no colonization at admission) who were tested positive for MDRO on either rectal screening or on a blood or respiratory sample were considered as having MDRO acquisition. Analyses comparing MDRO colonization were restricted to patients admitted to an ICU with systematic rectal colonization surveillance.

### Strategies for ICU-AI prevention

Strategies for pneumonia, CLABSI and BSI prevention were left at each ICU’s discretion. Most of the units use standard of care as recommended in the French intensivists recommendations [16, 17]. No measurement of compliance was made. In addition, six ICUs applied a selective decontamination strategy during the study period. In these ICUs, only mechanically ventilated patients with an expected length of mechanical support > 24 h received this prophylaxis. Details regarding selective decontamination strategy are reported in supplementary analysis and elsewhere [18].

### Primary and secondary endpoints

The primary endpoint was incidence rate of ICU-AI according to neutropenia at admission, while the secondary endpoints were specific incidence rates of pneumonia, BSI, CLABSI, but also ICU mortality, length of mechanical invasive ventilation, duration of ICU stay, presence of antibiotics within the 48 h. Finally, we aimed to describe the microbiology of ICU-AI according to neutropenia at admission.

Base on pertinent reviewers comments, three sensitivity analyses were conducted. First we conducted the analysis in a dataset in whom immunocompromised patients without neutropenia were excluded of the matching process. Then, considering the discussed pathogenicity of coagulase negative Staphylococcus sp. in BSI event, we conducted an analysis regarding BSI incidence in both groups after exclusion of cases in whom only a CNS was isolated. For explorating purpose, the case fatality rate for BSI event was reported. Finally, considering the debated pathogenicity of Enterococcus sp. in pneumonia, we conducted a sensitivity analysis, with exclusion of pneumonia in whom only Enterococcus spp. were identified.

### Ethics

The database was approved by the institutional review board (CPP SUD ESTdIRB 00009118) as well as by the National Data Protection Commission (Commission Nationale de l'Informatique et des Libertés, Number 919149). Specific information concerning this surveillance was given to all patients about the potential use of their personal data for research purposes.

### Statistical analysis

Statistical analysis was performed with the statistical software R 4.3.1. Categorical variables were expressed as percentages and continuous variables as median and interquartile range (IQR). The chi-square test and Fisher exact test were used to compare categorical variables and the Man-Whitney U test or the Wilcoxon for continuous variables.

To draw unbiased marginal estimates of exposure effect, a matching analysis was performed to evaluate the impact of neutropenia on outcomes (ICU-AI). Matching was calculated using non-parsimonious logistic regression model including all available baseline characteristics (i.e. age, sex, intubation status, central venous catheter, bladder catheter, modified simplified acute physiology score II (SAPS II) (since neutropenia is included in the simplified acute physiology score II, this component of the SAPS II score was deducted from the original SAPS II calculation to obtain the modified SAPS II calculation, used for propensity score calculation), antibiotherapy at admission, MRDO status at admission, admission in an ICU that applied selective decontamination, number of admission/bed/year in ICU of admission, diagnostic category (medicine, urgent surgery, scheduled surgery), trauma, COVID-19 status, department before for admission (home, acute care ward, long term care ward, other ICU), region of admission, year of admission). Using the “MatchIt” package, a k-nearest neighbor algorithm was used for propensity-score matching with a 1:1 ratio, using a caliper of 0.1. The balance between matched groups was evaluated by the analysis of the standardized mean differences after matching. A post-matching difference < 0.1 was considered as an optimal bias reduction.

Incidence rates were compared using a Poisson regression model. All tests were two-sided, and p < 0.05 was considered statistically significant.

## Results

### Settings

**Fig. 1:**
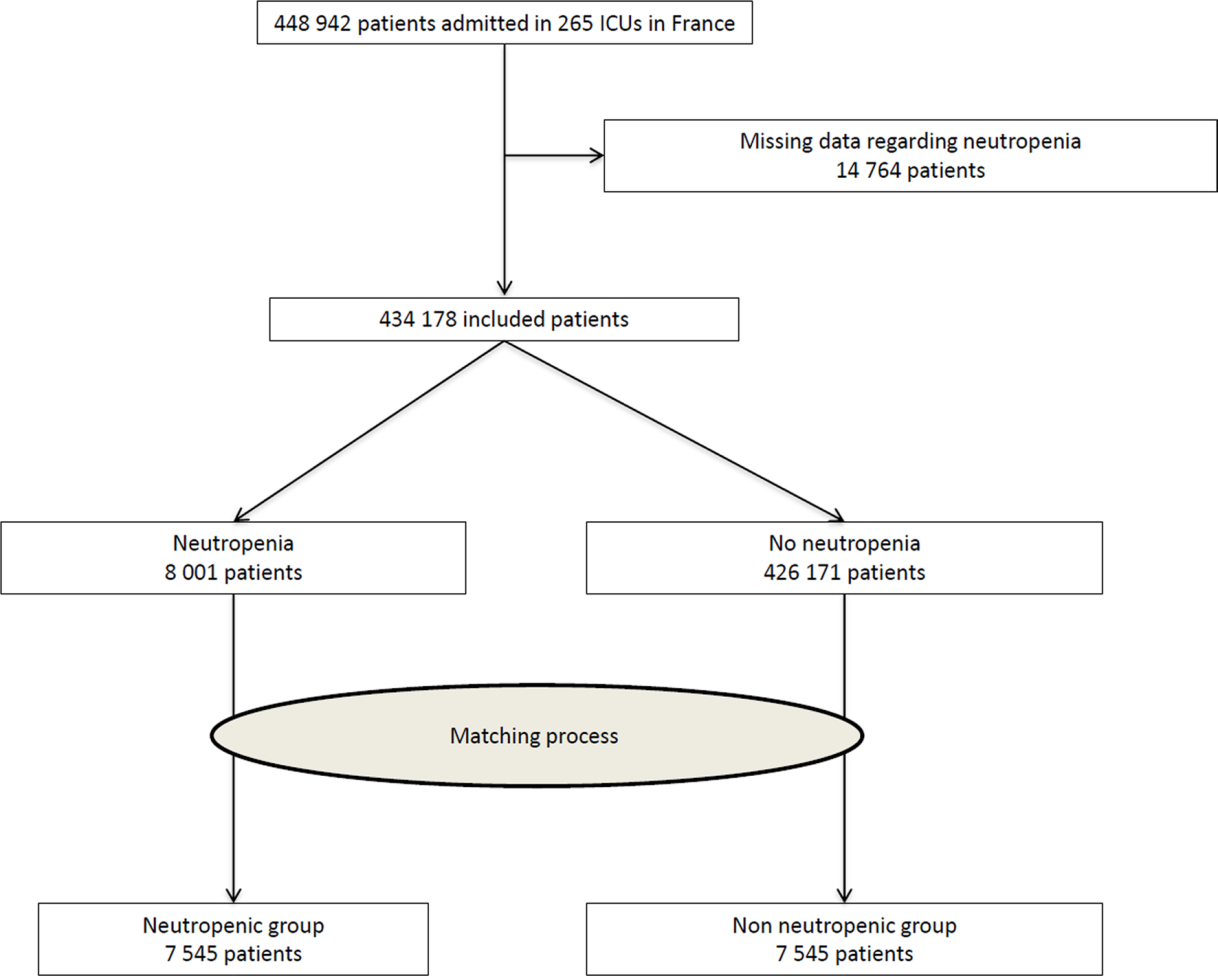
Flow chart

During the study period, 448 942 patients were admitted to the 265 participating ICUs. Among 434 178 included patients (14 764 were excluded because of data missing), 8001 patients were neutropenic, and 426 171 were non-neutropenic (Fig. 1).

### Complete population

The neutropenic group and the non-neutropenic group had slightly but significantly different turnover in ICU of admission with 28.62 [23.34,33.62] admission per bed per year vs 28.69 [23.45,34.06] respectively, (p = 0.002) and different HAS consumption L/patient/day 0.13 [0.09, 0.15] vs 0.12 [0.09, 0.16] respectively (p = 0.047). Others variables that differs between groups were antibiotherapy at admission, administrated to 6 537 (81.8%) patients in neutropenic group vs 241 291 (56.7%) patients in non-neutropenic group (p < 0.001), MDRO prevalence rate on admission rectal swab was observed in 132 patients (1.6%) in the neutropenic group as compared with 5 392 patients (1.3%) in the non-neutropenic group (p < 0.003), proportion of intubated patients with 3 928 (49.2%) intubated patients vs 257 793 (60.5%) intubated patients, respectively (p < 0.01) and finally proportion of patients admitted in an ICU that applied selective decontamination with 251 patients (6.4%) patients in neutropenic group vs 6 927 (2.7%) patients in non-neutropenic group (p < 0.01) (Supplementary Table 1).

During the study period, we identified 838 (10.5%) patients with ICU-AI including 543 (6.8%) with pneumonia, 437 (5.5%) with BSI and 45 (0.6%) with CLABSI in the neutropenic group as compared with 45 087 (10.6%) patients with ICU-AI including 36 421 (8.6%) with pneumonia, 15 627 (3.7%) with BSI and 1707 (0.4%) with CLABSI in non-neutropenic group (p < 0.001 for pneumonia and BSI and p = 0.030 for CLABSI; Supplementary Table 2).

Details on the features of patients are available in Supplementary Table.

### Matched patients pairs

Matching analysis resulted in a well-balanced study population that included 7 545 patients in both arms (Table 1).

**Table 1:** Baseline characteristics of study patients (matched patient pairs)

	Non-neutropenic patients	Neutropenic patients	p-value	
Variables	n = 7545	n = 7545		SMD
Age, year	64.40 [52.30, 74.30]	64.60 [54.80, 72.10]	0.488	0.02
Male—n (%)	4571 (60.6)	4623 (61.3)	0.395	0.01
SAPS II	42.00 [31.00, 56.00]	53.00 [41.00, 68.00]	< 0.001	-
Modified SAPS II*	42.00 [31.00, 56.00]	41.00 [29.00, 56.00]	0.070	0.01
Immunocompromised			< 0.001	-
	6224 (82.5)	0 (0.0)		
Others – n (%)	1321 (17.5)	0 (0.0)		
Neutropenia < 500 G/L—n (%)	0 (0.0)	7545 (100.0)		
Localization before admission			0.096	0.04
Other – n (%)	21 (0.3)	16 (0.2)		
Other ICU- n (%)	197 (2.6)	194 (2.6)		
Acute care ward- n (%)	4003 (53.1)	3864 (51.2)		
Home—(%)	3324 (44.1)	3471 (46.0)		
Trauma – n (%)	342 (4.5)	337 (4.5)	0.875	0.00
Type of admission			0.001	0.06
Planned surgery – n (%)	254 (3.4)	178 (2.4)		
Urgent surgery – n (%)	859 (11.4)	881 (11.7)		
Medicine – n (%)	6432 (85.2)	6486 (86.0)		
COVID-19—n (%)	208 (2.8)	219 (2.9)	0.623	0.01
Early management Therapeutic antibiotics – n (%)	6064 (80.4)	6218 (82.4)	0.001	0.05
Intubation – n (%)	3505 (46.5)	3663 (48.5)	0.010	0.04
Central venous catheter – n (%)	5687 (75.4)	5654 (74.9)	0.547	0.01
Bladder catheter – n (%)	6283 (83.3)	6288 (83.3)	0.930	0.00
MRDO colonisation at admission^$^ – n (%)	116 (2.4)	124 (2.6)	0.648	0.01
Selective decontamination in ICU – n (%)	91 (2.6)	82 (2.2)	0.359	0.01

ICU: Intensive-care unit. AI: Acquired Infection. COVID-19: SARS-COV 2 associated infection disease

^*^Since neutropenia is included in the simplified acute physiology score II, this component of the SAPS II score was deducted from the original SAPS II calculation to obtain the modified SAPS II calculation, used for propensity score calculation

^$^This variable was only evaluated for patients admitted in an ICU with systematic MDRO rectal carriage screening (4852 patients of the neutropenic group and 4851 patients of the non-neutropenic group)

Among matched patients pairs, there were 2 039 ICU-AI in 1 434 patients corresponding to 795 ICU-AI (10.5%) for neutropenic patients and 639 ICU-AI (8.5%) for non-neutropenic patients (p < 0.001) (Table 2).

**Table 2:** Outcomes (matched patient pairs)

	Non-Neutropenic patients	Neutropenic patients	p
Variables	n = 7545	n = 7545	
AI—n (%)	639 (8.5)	795 (10.5)	< 0.001
Time to first AI for patients with AI, days	9.00 [5.00, 15.00]	9.00 [5.00, 15.00]	0.236
Site of ICU-AI Pneumonia – n (%)	465 (6.2)	511 (6.8)	0.139
BSI—n (%)	260 (3.4)	420 (5.6)	< 0.001
CLABSI = 1 (%)	29 (0.4)	45 (0.6)	0.080
MRDO AI—n (%)	98 (15.3)	137 (17.2)	0.372
MRDO acquisition^$^—n (%)	61 (1.3)	54 (1.1)	0.573
Length of mechanical ventilation for intubated patients, days	5.00 [2.00, 11.00]	6.00 [2.00, 12.00]	< 0.001
Lenght of stay in ICU, days	6.00 [3.00, 10.00]	6.00 [3.00, 11.00]	< 0.001
Death in the ICU—n (%)	1197 (15.9)	2000 (26.5)	< 0.001

ICU: Intensive-care unit. AI: Acquired Infection. MDRO: Multi Drug Resistant Micro Organisms. MDRO AI: Multi Drug Resistant Micro Organisms Aquired infection. BSI = blood steam infection. CLABSI: catheter line-associated blood stream infection

^$^This variable was only evaluated for patients admitted in an ICU with systematic MDRO rectal carriage screening (4852 patients of the neutropenic group and 4851 patients of the non-neutropenic group)

In patients with ICU-AI, time from admission to first ICU-AI, was not different between groups (9 days [5–15] in both groups, p = 0.236). Patients in the neutropenic group had a longer duration of invasive mechanical ventilation than those in the non-neutropenic group with respectively 6 days [2.00, 2.00] vs 5 days [2.00, 11.00] (p < 0.001). The length of stay and ICU mortality were higher in the neutropenic group than in the non-neutropenic group with respectively 6 days [3.00, 11.00] vs 6 days [3.00, 10.00] (p < 0.001) of length of stay, and 2000 ICU death (26.5%) vs 1197 ICU death (15.9%), respectively (p < 0.001) (Table 2). ICU-AI incidence rate was 14.9 per 1000 patients days in the neutropenic group versus 13.0 ICU-AI per 1000 patients days in the non-neutropenic group (Incidence Rate Ratio [IRR] = 1.15 [1.05, 1.25] p = 0.002). BSI incidence rate was 6.29 per 1000 patients days as compared with 4.53 per 1000 patients days, in the neutropenic and non-neutropenic groups, respectively (IRR = 1.39 [1.21, 1.60] p < 0.001). When BSI whose sole causative agent were coagulase-negative Staphylococci were removed from the analysis, patients in the neutropenic group still had a higher incidence of BSI (IRR = 1.34 [1.16, 1.55] p < 0.001). Interestingly, neutropenic patients who developed a BSI involving coagulase-negative Staphylococci had a higher mortality rate than those who had no BSI (45% vs 25%, respectively, p < 0.001) and case fatality rate of patients with coagulase-negative Staphylococci BSI was close to those of patients with BSI involving *enterobacteraless* (45% vs 43%, respectively, p = 0.73) (Supplementary Table 3).

Pneumonia incidence rate was 8.0 per 1,000 patient-days in neutropenic patients compared with 10.9 per 1,000 patient-days in non-neutropenic patients, yielding an incidence rate ratio of 0.74 (95% CI, 0.69–0.80; p < 0.0001). VAP rate was 16.7 per 1000 invasive mechanical ventilation days as compared with 17.4 per 1000 invasive mechanical ventilation days, respectively (IRR = 0.96 [0.86, 1.08] p = 0.503). Given that the pathogenicity of Enterococcus in the lung remains uncertain, we conducted a secondary analysis, with exclusion of pneumonia in whom only Enterococcus species were identified. The incidence rate of pneumonia was not statistically different in this analysis neither (IRR = 0.93 [0.82, 1.05] p = 0.241).

Finally, the incidence of CLABSI was 0.66 per 1,000 patient-days in the neutropenic group compared with 0.46 per 1,000 patient-days in the non-neutropenic group (IRR = 1.46; 95% CI, 0.93–2.29; p = 0.100).

We then conducted a sensitivity analysis with exclusion of non-neutropenic immunocompromised patients. The dataset consisted of 7,545 neutropenic patients matched with 7,545 patients without any immunodeficiency. In this dataset, ICU-AI incidence was similar between groups (IRR 1.07, 95% CI 0.97–1.15; p = 0.170) while the risk of pneumonia was lower in neutropenic patients (IRR 0.89, 95% CI 0.80–0.99; p = 0.043), and the risk of bloodstream infection significantly higher (IRR 1.39, 95% CI 1.12–1.60; p < 0.001).

### Microbiology

**Fig. 2:**
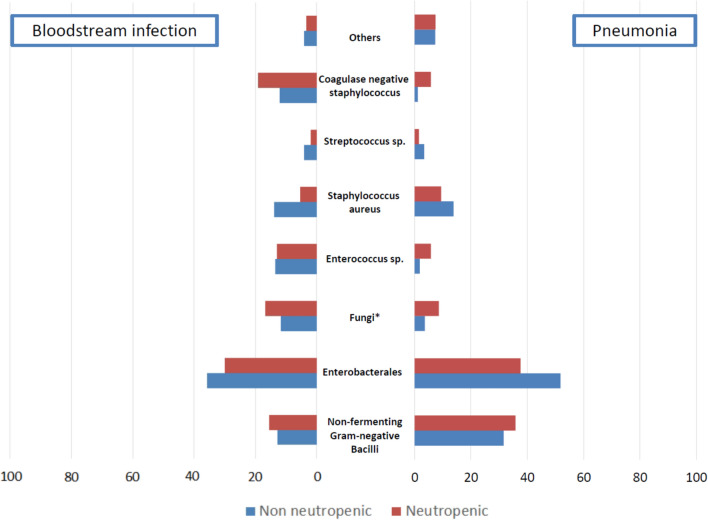
Microorganism involved in Pneumonia and Bloodstream infection in both groups. Notes. *Fungi corresponded to Aspergillus sp. in all pneumonia cases (22 in the neutropenic group and 6 in the non-neutropenic group) and candida sp. in all BSI cases (118 in the neutropenic group and 56 in the non-neutropenic group)

Data on organisms responsible for ICU-AI are reported by site of infection in Table 3 and Fig. 2.

**Table 3:** Data regarding AI (matched patient pairs)

	Non-neutropenic patients	Neutropenic patients	p
Variables	n = 907	n = 1132	
Non-fermenting Gram-negative Bacilli—n (%)	224 (24.7)	302 (26.7)	0.334
Enterobacterales—n (%)	412 (45.4)	383 (33.8)	< 0.001
Candida—n (%)	56 (6.2)	118 (10.4)	0.001
Enteroccocus sp.—n (%)	54 (6.0)	101 (8.9)	0.015
Staphyloccocus aureus—n (%)	131 (14.4)	88 (7.8)	< 0.001
Streptoccocus sp.—n (%)	32 (3.5)	18 (1.6)	0.008
Coagulase negative staphyloccocus—n (%)	47 (5.2)	136 (12.0)	< 0.001
Virus—n (%)	5 (0.6)	11 (1.0)	0.414
Aspergillus sp. – n (%)	6 (0.7)	22 (1.9)	0.023
Anaerobic—n (%)	13 (1.4)	17 (1.5)	1.000
Others—n (%)	41 (4.5)	53 (4.7)	0.947
MRDO AI—n (%)	110 (13.6)	126 (12.7)	0.586
Type of MRDO			0.407
ESBL- PE – n (%)	57 (51.8)	69 (54.8)	
CRE—n (%)	11 (10.0)	13 (10.3)	
VRE—n (%)	0 (0.0)	2 (1.6)	
MDR NF-GNB – n (%)	21 (19.1)	27 (21.4)	
MRSA – n (%)	21 (19.1)	15 (11.9)	
Bloodstream infection	n = 316	n = 478	p
Non-fermenting Gram-negative Bacilli—n (%)	40 (12.7)	74 (15.5)	0.314
Enterobacterales—n (%)	113 (35.8)	143 (29.9)	0.100
Candida—n (%)	37 (11.7)	80 (16.7)	0.064
Enteroccocus sp.—n (%)	43 (13.6)	62 (13.0)	0.879
Staphyloccocus aureus—n (%)	44 (13.9)	26 (5.4)	< 0.001
Streptoccocus sp.—n (%)	13 (4.1)	9 (1.9)	0.098
Coagulase negative staphyloccocus—n (%)	38 (12.0)	92 (19.2)	0.009
Anaerobic—n (%)	13 (4.1)	16 (3.3)	0.711
MDRO-AI—n (%)	28 (9.4)	48 (10.7)	0.642
Pneumonia	n = 560	n = 606	p
Non-fermenting Gram-negative Bacilli—n (%)	177 (31.6)	217 (35.8)	0.146
Enterobacterales—n (%)	290 (51.8)	228 (37.6)	< 0.001
Enteroccocus sp.—n (%)	10 (1.8)	35 (5.8)	0.001
Staphyloccocus aureus—n (%)	77 (13.8)	57 (9.4)	0.026
Streptoccocus sp.—n (%)	19 (3.4)	9 (1.5)	0.053
Coagulase negative staphyloccocus—n (%)	6 (1.1)	35 (5.8)	< 0.001
Virus—n (%)	4 (0.7)	9 (1.5)	0.330
Aspergillus sp.– n (%)	6 (1.1)	22 (3.6)	0.008
Others—n (%)	55 (9.8)	75 (12.4)	1.000
MDRO-AI—n (%)	80 (16.5)	74 (14.4)	0.414
CLABSI	n = 31	n = 48	
Non-fermenting Gram-negative Bacilli—n (%)	7 (22.6)	11 (22.9)	1.000
Enterobacterales—n (%)	9 (29.0)	12 (25.0)	0.892
Enteroccocus sp.—n (%)	1 (3.2)	4 (8.3)	0.662
Staphyloccocus aureus—n (%)	10 (32.3)	5 (10.4)	0.034
Coagulase negative staphyloccocus—n (%)	3 (9.7)	9 (18.8)	0.438
Candida sp. – n (%)	5 (16.1)	8 (16.7)	1.000
Others—n (%)	0 (0.0)	1 (2.1)	1.000
MDRO-AI—n (%)	2 (9.5)	4 (12.5)	1.000

ICU: Intensive-care unit. AI: Acquired Infection. ESBL-PE: extended spectrum beta lactamase producing *Enterobacterales*. MDRO: Multi Drug Resistant Micro Organisms. MDRO NF-GNB: multi drug resistant organisms Non fermenting – gram negative bacilli. CRE: Carbapenem resistant *Enterobacterales.* VRE: Vancomycin resistant *Enterococcus sp.* MRSA: Methicillin Resistant *Staphylococcus aureus.* CLABSI: catheter line-associated blood stream infection

Among those with ICU-AI, 32.1% of non-neutropenic patients had polymicrobial ICU-AI vs. 31.7% of neutropenic patients (p = 0.922).

The distribution of organisms responsible for ICU-AI differed in between groups, with a lower proportion of *Enterobacterales* in the neutropenic group as compared with the non-neutropenic group (33.8% vs 45.4%, respectively, p < 0.001), especially in pneumonia (37.6% vs 51.8%, respectively, p < 0.001). There were a higher proportion of coagulase negative *Staphylococci* (12.0% vs 5.2%, p < 0.001), in the ICU-AI of the neutropenic group, especially in BSI. Similarly, *Candida spp.* (10.4% vs 6.2%, respectively, p = 0.001) were more frequent in ICU-AI of the neutropenic group, as were *Enterococcus spp.* (8.9% vs 6.0%, respectively, p = 0.015).

Filamentous fungi were more also frequently identified in ICU-AI of the neutropenic group, specifically in pneumonia cases (3.6% vs 1.1%, respectively p = 0.008). Finally, there were no difference in rates of ICU-AI involving non-fermenting Gram-negative *bacilli* (26.7% vs 24.7%, respectively; p = 0.334).

MDRO colonization acquisition occurred in 54 (1.1%) patients of the neutropenic group and 61 (1.3%) patients in the non-neutropenic group (p = 0.573).

Among patients with ICU-AI, MDRO were observed in 126 ICU-AI in the neutropenic group as compared with 110 in the non-neutropenic group (12.7% vs 13.6% of ICU-AI in each group, respectively, p = 0.586). The first mechanism of antimicrobial agent resistance in both groups was extended spectrum beta-lactamase producing *Enterobacterales* (Table 3).

## Discussion

In this nationwide cohort study, we observed that neutropenic patients admitted in French ICUs had higher ICU-AI incidence rate than non-neutropenic patients, specifically through an increase of BSI incidence rate while pneumonia and CLABSI incidence rates were similar. The microbiology of ICU-AI was also different between both groups but the rate of MDRO related AI and MRDO colonization acquisition were similar in both groups.

Recent investigations have challenged the common assumption that immunocompromised patients are at higher risk of ICU-AI infections [3, 19]. Neutropenia can cause damage to the intestinal mucosa, making it more vulnerable to intramural bacterial invasion [20, 21]. Nevertheless, the impact of intensive care on the risk of bloodstream infection in immunocompromised patients remains uncertain, with studies reporting divergent findings. In this study, we found higher rate of BSI in the neutropenic group. Few studies have specifically investigated the association between all causes of immunosuppression and the incidence of ICU- acquired BSI. Previously, some authors suggested that all-cause immunosuppression at ICU admission was not a risk factor for ICU-acquired BSI [22–24]. In the COCONUT study [24] aimed to assess whether immunosuppression at ICU admission was associated with the 28-day incidence of ICU-acquired bacterial bloodstream infections, immunosuppression was not associated with a higher incidence of ICU‐ acquired BSI, but in this study, the main cause of immunosuppression was the use of immunosuppressive therapies, and only a sixth of included patients were neutropenic.

Importantly, occurrence of ICU acquired BSI had been associated with increased mortality in unselected critically patients. In the EUROBACT study [12], Tabah et al. found that among patients with hospital-acquired BSI (three quarter being acquired in the ICU), immunosuppression was associated with an increased mortality.

Finally, in the study presented here, there was no significant difference between rates of pneumonia in neutropenic group versus non-neutropenic group. The modified results observed in the sensitivity analysis underscore the heterogeneity of immunocompromised populations and suggest that neutropenic and non-neutropenic patients may have distinct infectious risk profiles. In an ancillary analysis of a prospective multicenter observational study, the proportion of pneumonia was significantly lower among immunocompromised than among non-immunocompromised patients, and in a retrospective multicenter analysis focusing only on immunocompromised patients, the incidence of VA-LRTI was lower among patients with hematologic malignancies than among patients with other types of immunosuppression [4, 25]. The proportion of intubated patients was higher in non-neutropenic group. Nevertheless, pneumonia incidence rate based on invasive mechanical ventilation days did not differ between both groups. VA-LRTI diagnosis was based on a body of argument, some of which may be absent in neutropenic patients (*i.e.* leucocytosis, purulent sputum, fever). Consequently, VA-LRTI incidence might have been underestimated in neutropenic patients.”

Specific data on the microbiology of ICU-acquired infections in immunocompromised patients are scarce [20], particularly for neutropenic patients. Here, in neutropenic group, *Enterobacterales* was the main microorganisms identified followed by non-fermenting Gram-negative bacilli and coagulase negative S*taphylococcus, Candida spp* and *Enterococcus spp*. The distribution of organisms responsible for AI differed with non-neutropenic patients. Interestingly, there were a higher proportion of coagulase negative *Staphylococci, Enterococcus spp,* and *Candida spp* in neutropenic patients. *Candida spp* and *Enterococcus spp* belong to the commensal flora of the digestive tract. Changes in the ecology of the resident microbiome, such as those induced by the administration of broad-spectrum antibiotics, favor the growth of these species, which then colonize mucosal surfaces. When mucosal integrity is compromised, as in neutropenia, invasive infection can occur. Furthermore, it should be emphasized that nosocomial transmission can occur via exogenous transmission. In our study, a higher proportion of patients in the neutropenic group had a central venous catheter. This could partly explain the higher number of Candida and coagulase negative *Staphylococci* infections. Surprisingly, the proportion of infection involving Enterobacterales was lower in neutropenic patients, especially in pneumonia. Similarly, there were less ICU-AI involving *Staphylococcus aureus* in the neutropenic group. The proportion of patients receiving antibiotics in both groups was close after matching. However, since we had no data regarding the molecule administrated, it is possible that neutropenic patients received more likely an antibiotic that targeted Enterobacterales or *Staphylococcus aureus*, thus decreasing the risk of subsequent infection.

Furthermore, here, microbiological documentation was performed by positive culture on respiratory samples (tracheal aspiration or bronchoalveolar lavage) and rapid multiplex PCR tests were not considered, even though they offer increased speed and sensitivity particularly in immunocompromised population [26]. Among the patients with AI, surprisingly, there were as many MDRO in neutropenic group than non-neutropenic group and MDRO colonization acquisition occurred similarly in both groups. In the COCONUT study [24], among the bacteria responsible for BSI, Gram-negative bacilli were the most frequent organisms identified, mainly *Klebsiella pneumoniae* and *Enterobacter* spp*.*, followed by Gram-positive cocci, mainly coagulase-negative *staphylococci* and the distribution of bacteria was comparable between groups. The proportion of ICU acquired BSI related to MDR bacteria was comparable between groups.

Recent data have shown that the cumulative incidence of ICU-acquired infections with MDRO bacteria might be lower in immunocompromised patients than non-immunocompromised ones [25]^.^ In a prospective multicentric study, after adjustment for pre-specified baseline confounders (previous antibiotherapy, hospital stay), immunocompromised patients had a lower incidence rate ICU-MDRO colonization but similar incidence rate ICU MDRO AI. The distribution of MDRO bacteria was comparable between groups [19]. In a multicenter cohort, antimicrobial resistance patterns were comparable between immunocompromised and non-immunocompromised patients, including similar rates of multidrug resistance (19.6% vs. 15.0%, p = 0.27), ESBL-producing strains (8.8% vs. 8.9%, p = 0.74), carbapenemase-resistant isolates (8.8% vs. 6.2%, p = 0.33), and MRSA (5.6% vs. 7.0%, p = 0.39) [27]. In fact, the emergence of resistance is multifactorial and probably depends on the global use of antibiotics during the entire patient's hospital stay.

Mortality rate was higher in neutropenic patients, a difference that could not be explained solely by the higher ICU-AI incidence rate observed. Similarly, in the coconut study, the author reported a higher mortality in immunocompromised patients. They observed that immunosuppression at ICU admission had no effect on the association between occurrence of BSI and patient outcomes [24].

The results presented here pertain specifically to neutropenic patients admitted to the ICU and should not be generalized to all immunocompromised patients, who constitute a highly heterogeneous population with diverse characteristics and clinical trajectories. Given that results differed in the sensitivity analysis, these findings must be interpreted with caution. They suggest that the inclusion of non-neutropenic immunocompromised patients may have influenced the primary analysis and underscore the importance of future studies focusing on more homogeneous subgroups.

## Limitations

The participation of a very large number of French ICU has enabled us to obtain recent information on a very large volume of neutropenic patients admitted to intensive care over the last decade. However, these results may not be generalizable to all intensive care units, given the variability in patient recruitment and local microbial ecology across centers.

Nevertheless, due to observational design, we were unable to obtain details on the history of neutropenia. Information concerning the etiology of neutropenia was not available even though the depth of immunosuppression should vary according to the cause of neutropenia. In addition, its duration prior to admission to the ICU was not available. Then, uncontrolled variables, may also have affected our findings.

Over the past few years, advancements in onco-hematologic therapies have significantly altered the landscape of patient management, leading to dynamic and heterogeneous patterns of immunosuppression. These evolving immunocompromised phenotypes differed substantially between the initiation of data collection in 2018 and its completion in 2023. It should be noted, however, that no formal comparative analysis of study endpoints was conducted between the early and late phases of the surveillance period.

Furthermore, as underlined above, ICU-AI diagnosis might be considered as a subjective endpoint as it was not reviewed by independent experts. Diagnosis methods might differ between centers. Moreover, ICU-AI might present differently in neutropenic patients as compared with immunocompetent ones. Specifically, ICU acquired pneumonia diagnosis relies on signs that are frequently absent in neutropenic patients (i.e. leucocytosis, purulent sputum, fever). Then, different time to positivity of blood culture was not collected in our study. However, its performance varies according with the microorganism involved in CLABSI and has disputable diagnostic performance [28].

Finally, at present, there is no specific definition or diagnosis method for ICU-AI in immunocompromised.

## Conclusions

Our observational study suggested that neutropenic patients have higher rate of AI, mostly due to a higher BSI incidence rate. Microbiology differs between neutropenic patients and non-neutropenic patients with higher proportion of coagulase negative *Staphylococcus, Enterococcus spp,* and *Candida spp* in neutropenic patients, while there were similar proportion of AI involving MDRO. Our results suggest that the clinical management of ICU-AI might require a specific approach in neutropenic patients, especially regarding the higher risk of BSI and the specificity of its epidemiology. Further study that aims to evaluate infection prevention measure in this specific setting should focus on this specific microorganism and on BSI prevention in order to decrease AI related mortality and morbidity.

### Take home message

- Neutropenic patients had higher AI incidence rate than non-neutropenic patients, specifically through an increase of BSI incidence rate while pneumonia and CLABSI incidence rates were similar.
- Microbiology differs between neutropenic patients and non-neutropenic patients with higher proportion of coagulase negative *Staphylococcus, Enterococcus *spp, and *Candida* spp in neutropenic group.
- There were no increase of MRDO related AI nor increased MRDO colonization acquisition among neutropenic patients.

## Supplementary Information

Additional file 1

## Data Availability

The datasets generated during the current study are available from the corresponding author on reasonable request.
