# Supplementary material for: ICU-acquired infection in neutropenic patients
Source: Crit Care. 2026 Mar 17;30:205. doi: 10.1186/s13054-026-05890-5 (PMC13113094; doi:10.1186/s13054-026-05890-5)
Supplement: Supplementary file 1 — Additional file 1 [file 13054_2026_5890_MOESM1_ESM.docx]

**Supplementary table 1. Baseline characteristics.**

| Variables | Non-neutropenic | Neutropenic | p |
| --- | --- | --- | --- |
|  | 426176 | 8001 |  |
| Age, year | 66.80 [55.60, 76.00] | 64.70 [54.50, 72.10] | <0.001 |
| Male sex – n (%) | 269081 (63.1) | 4921 (61.5) | 0.003 |
| SAPSII | 43.00 [31.00, 57.00] | 53.00 [41.00, 68.00] | <0.001 |
| Number of admission per bed per year | 28.69 [23.45, 34.06] | 28.62 [23.34, 33.62] | 0.021 |
| Prepandemic period – n (%) | 321955 (75.5) | 5980 (74.7) | 0.100 |
| Hand sanitizing consumption, L per patient-day | 0.12 [0.09, 0.16] | 0.13 [0.09, 0.15] | 0.470 |
| Covid-19 - n (%) | 18555 (4.4) | 238 (3.0) | <0.001 |
| Early management  Antibiotherapy at admission - n (%) | 241291 (56.7) | 6537 (81.8) | <0.001 |
| Intubation - n (%) | 257793 (60.5) | 3928 (49.2) | <0.001 |
| Central veinous catheter - n (%) | 283307 (66.6) | 5972 (74.8) | <0.001 |
| Bladder catheter – n %) | 359730 (85.5) | 6418 (83.3) | <0.001 |
| MRDO – n (%) | 5392 (1.3) | 132 (1.6) | 0.003 |
| Admission in an ICU that applied Selective decontamination - n (%) | 6927 (2.7) | 251 (6.4) | <0.001 |

Notes. ICU : Intensive-care unit. AI : Acquired Infection. COVID-19 : SARS-COV 2 associated infection disease. MRDO : multi drug resistant organism.

**Supplementary table 2. Outcomes.**

|  | Non-neutropenic | Neutropenic | p |
| --- | --- | --- | --- |
| n | 426176 | 8001 |  |
| AI - n (%) | 45087 (10.6) | 838 (10.5) | 0.775 |
| Time to first AI, days | 8.00 [5.00, 14.00] | 9.00 [5.00, 14.00] | 0.621 |
| Site of ICU-AI  Pneumonia - n (%) | 36421 (8.6) | 543 (6.8) | <0.001 |
| BSI - n (%) | 15627 (3.7) | 437 (5.5) | <0.001 |
| CLABSI – n (%) | 1707 (0.4) | 45 (0.6) | 0.030 |
| MRDO AI – n (%) | 7170 (15.9) | 149 (17.8) | 0.154 |
| MRDO acquisition - n (%) | 4052 (1.0) | 57 (0.7) | 0.034 |
| Lenght of mechanical ventilation, days | 4.00 [1.00, 10.00] | 6.00 [2.00, 12.00] | <0.001 |
| Lenght of ICU stay, days | 5.00 [3.00, 11.00] | 6.00 [3.00, 11.00] | <0.001 |
| Death - n (%) | 72264 (17.0) | 2085 (26.1) | <0.001 |

Notes. ICU : Intensive-care unit. AI : Acquired Infection. MDRO : Multi Drug Resistant Micro Organisms. MDRO AI : Multi Drug Resistant Micro Organisms Acquired infection. BSI : blood stream infection. CLABSI : catheter line-associated blood stream infection.

**Supplementary table 3.** Case fatality rate for patients with BSI, according with the microorganism involved in BSI event.

| Micro-organism responsible for BSI | Mortality rate  Number of death/Number of event (%) | |  |
| --- | --- | --- | --- |
|  | Non-neutropenic | Neutropenic | p |
| Non-fermenting Gram-negative Bacilli | 14/40 (35) | 45/73 (62) | 0.007 |
| Enterobacteriaceae | 25/102 (25) | 59/138 (43) | 0.003 |
| Candida | 16/35 (46) | 53/78 (68) | 0.025 |
| Enteroccocus sp. | 11/41 (27) | 36/60 (60) | 0.001 |
| Staphyloccocus aureus | 10/42 (24) | 9/25 (36) | 0.28 |
| Streptoccocus sp. | 6/13 (43) | 5/9 (35) | 0.66 |
| Coagulase negative staphyloccocus | 12/37 (32) | 37/82 (45) | 0.193 |
| Anaerobic | 1/13 (8) | 9/16 (56) | 0.006 |
| MDRO-AI | 5/26 (19) | 22/47 (47) | 0.019 |
| No BSI | 1121/7280 (15) | 1781/7125 (25) | <0.001 |

Notes. MDRO : Multi Drug Resistant Micro Organisms. MDRO AI : Multi Drug Resistant Micro Organisms Acquired infection. BSI : blood stream infection.
